# ALK融合阳性非小细胞肺癌新辅助靶向治疗的进展

**DOI:** 10.3779/j.issn.1009-3419.2024.106.30

**Published:** 2024-11-20

**Authors:** Weizhen SUN, Yuheng ZHOU, Yaobin LIN, Shoucheng FENG, Hao LONG

**Affiliations:** 510000 广州，中山大学肿瘤防治中心胸外科，华南肿瘤学国家重点实验室; Department of Thoracic Surgery, Sun Yat-Sen University Cancer Center, State Key Laboratory of Oncology in South China,Guangzhou 510000, China

**Keywords:** 肺肿瘤, ALK融合阳性, 新辅助治疗, 靶向治疗, Lung neoplasms, ALK-positive, Neoadjuvant therapy, Targeted therapy

## Abstract

肺癌仍然是世界范围内最常见的癌症和癌症相关死亡的主要原因，其中间变性淋巴瘤激酶（anaplastic lymphoma kinase, ALK）融合突变所占的比例为4%-9%。近年来，越来越多的临床证据表明ALK抑制剂与手术治疗相结合的治疗模式在可切除的早期与局部晚期非小细胞肺癌（non-small cell lung cancer, NSCLC）患者中存在着较大的临床应用潜力。本综述旨在总结ALK融合阳性NSCLC新辅助靶向治疗的研究进展，并分析其在临床实践中的优势与挑战。

## Abstract (en)

Lung cancer remains the most frequently diagnosed cancer and the leading cause of cancer-related death worldwide, with anaplastic lymphoma kinase (ALK) fusion mutations accounting for approximately 4%-9% of cases. In recent years, there are increasing clinical evidences suggesting that the combination of ALK inhibitors with surgical treatment holds significant potential for clinical application in resectable early and locally advanced non-small cell lung cancer (NSCLC) patients. This review aims to summarize the advances in neoadjuvant targeted therapy for ALK fusion positive NSCLC and discuss its advantages and challenges in clinical practice.

肺癌目前仍是全球范围内最常见的恶性肿瘤，也是最主要的癌症致死原因，其中非小细胞肺癌（non-small cell lung cancer, NSCLC）占所有肺癌的80%-85%^[1]^。目前，根治性手术被认为是NSCLC患者唯一获得疾病治愈可能的治疗选择，但该部分患者仅占到所有初诊患者的30%，其中位5年总体生存（overall survival, OS）率仅为36%-60%^[2]^。因此，随着靶向治疗与免疫治疗药物的发展，以手术为中心的综合性治疗模式被认为是提高早中期与局部晚期NSCLC患者长期生存的重要手段。目前，围手术期免疫治疗已被多项国际多中心临床研究（如KEYNOTE-671^[3]^、AEGEAN^[4]^、CheckMate-816^[5]^等）证实可有效提高病理学缓解程度、根治性手术切除率，降低疾病复发风险以及提高OS率。然而，围手术期免疫治疗对于存在驱动基因突变[如表皮生长因子受体（epidermal growth factor receptor, EGFR）、间变性淋巴瘤激酶（anaplastic lymphoma kinase, ALK）、c-ros肉瘤致癌因子-受体酪氨酸激酶（ROS proto-oncogene 1, receptor tyrosine kinase, ROS1）等]患者的治疗效果不尽如人意，而围手术期靶向治疗的安全性与有效性仍处于小范围的探索阶段。

ALK是肺癌的核心驱动基因之一。ALK融合阳性患者占NSCLC患者的4%-9%^[6]^，因ALK酪氨酸激酶抑制剂（tyrosine kinase inhibitors, TKIs）靶向治疗在晚期NSCLC患者中显著的生存获益而被称为“钻石突变”。CROWN研究5年的结果^[7]^中洛拉替尼组在中位随访时间为60.2个月时仍未达到中位无进展生存期（progression-free survival, PFS）。PROFILE 1014和ASCEND-4研究^[8,9]^表明，与铂类化疗相比，使用ALK-TKIs治疗未经治疗的晚期ALK融合阳性NSCLC患者具有明显的生存获益，其PFS明显延长。ALINE研究^[10]^表明ALK-TKIs对于NSCLC患者术后辅助治疗也有着良好的疗效，阿来替尼组2年无病生存率（disease free survival, DFS）为93.8%。

因此，随着ALK-TKIs在晚期以及接受根治性手术后的NSCLC患者中的治疗疗效愈发成熟与确切，越来越多的研究开始探索ALK-TKIs用于早中期与局部晚期患者新辅助靶向治疗的临床价值。本文总结了当前ALK融合阳性NSCLC新辅助靶向治疗的研究进展，探讨其在临床实践中的应用和潜力，并讨论其目前的局限性与挑战。

## 1 ALK融合与ALK-TKIs

ALK融合是指ALK发生断裂并与其他基因融合，翻译后ALK融合蛋白构象改变，影响自身磷酸化而导致肿瘤发生，成为肿瘤的驱动基因^[11]^。棘皮动物微管相关蛋白样4（echinoderm microtubule-associated protein-like 4, EML4）-ALK是NSCLC中最常见的ALK融合，约在85%的病例中发现^[12]^，但ALK也与EML4以外的伴侣基因发生重排，包括KIF5B、KLC1、TFG、TPR、HIP1、STRN、DCTN1、SQSTM1、NPM1、BCL11A和BIRC6等90多个不同的伴侣基因，并且已发现的所有ALK基因融合突变都是在ALK基因外显子20处发生断裂^[13]^。

EML4-ALK通过ALK融合蛋白激活PI3K-AKT、RAS和JAK/STAT信号通路从而影响肿瘤的进展、生存和生长，ALK-TKIs通过抑制ALK融合蛋白磷酸化来发挥抗肿瘤作用^[14,15]^。ALK-TKIs均为竞争性三磷酸腺苷（adenosine triphosphate, ATP）抑制剂，第一代与第二代TKIs仅在靶点敏感性、有效性方面有所区别，而第三代TKIs在结构上与前两代有明显差异。第三代TKIs具有大环酰胺结构，结构上更加紧凑，与药物结合作用力更强，从而提升了抗肿瘤活性，克服多种ALK耐药突变^[16]^。目前国内批准上市的ALK-TKIs共计7款，第一代ALK-TKIs有克唑替尼（Crizotinib），第二代ALK-TKIs有塞瑞替尼（Ceritinib）、阿来替尼（Alectinib）、恩沙替尼（Ensartinib）、布加替尼（Brigatinib）和伊鲁阿克（Iruplinalkib），第三代ALK-TKIs有洛拉替尼（Lorlatinib）。目前在研第四代ALK靶向药有康太替尼（Conteltinib）、复瑞替尼（Foritinib）、TGRX-326及SY-3505等。

## 2 ALK融合阳性NSCLC新辅助治疗进展

迄今为止，已有包括克唑替尼、阿来替尼和塞瑞替尼等在内的多款ALK-TKIs在新辅助靶向治疗领域中进行了探索。

2018年Zhang等^[17]^回顾性分析了11例初诊为N2期可切除的局部晚期ALK融合阳性NSCLC患者接受克唑替尼新辅助治疗的疗效及安全性，其中10例（91%）患者在影像学上取得部分缓解（partial response, PR），1例（9%）患者表现为疾病稳定（stable disease, SD），所有患者在新辅助克唑替尼治疗后均接受了手术，其中10例（91%）接受了完全（R0）切除术且围手术期未出现明显的手术并发症，20%患者达到了病理学完全缓解（pathological complete response, pCR）。在后续的随访过程中共6例患者出现了疾病复发，其中位DFS为10.1个月。该研究首次证实了ALK-TKIs在新辅助治疗中的可行性，并显示出良好的耐受性与潜在的临床获益。然而，鉴于该研究为一项回顾性研究，研究者未能对患者接受新辅助靶向治疗的时间进行标准化控制，11例患者的治疗时间跨度较大，从28-120 d不等，因此其所报告的病理学缓解水平难以衡量。此外，该队列患者术后接受的辅助治疗方案亦不相同，各自分别接受了包括辅助化疗、放化疗以及克唑替尼治疗等在内的多种治疗方案，导致难以评估新辅助靶向治疗对于降低术后复发风险的疗效。而且，关于ALK-TKIs新辅助治疗的多项关键问题仍待深入探讨，包括最佳的新辅助治疗时长、手术的最佳时机以及术后辅助治疗方案的选择。未来的研究需提供更多的证据来解决这些问题，以便为临床实践提供指导。

另一项前瞻性研究由Zenke等^[18]^于2019年报道，这是一项多中心、单臂、II期的临床研究（SAKULA），该研究共纳入了7例IIIA期ALK融合阳性NSCLC患者，在手术前共进行3个周期（共计12周）的塞瑞替尼新辅助治疗。6例患者在治疗后接受了手术治疗，其中根治性切除（R0）率为83.3%（5/6）。术后病理报告提示，共4例（57%）患者获得了主要病理学缓解（major pathological response, MPR），其中2例（29%）患者达到了pCR。该结果表明，新辅助塞瑞替尼在ALK融合阳性可切除NSCLC患者中是安全有效的，且具有较高的病理反应率。

在2023年美国胸外科学会（American Association for Thoracic Surgery, AATS）年会上，Zhang等^[19]^汇报了一项针对我国患者的ALK-TKIs围手术期治疗IIIA-IIIB期ALK融合阳性NSCLC的回顾性对照研究，研究中纳入39例接受阿来替尼或克唑替尼治疗的患者，29例患者在接受ALK-TKIs治疗后进行了手术，均行R0切除术。与克唑替尼相比，阿来替尼在MPR（56.3% vs 30.8%）和pCR（37.5% vs 15.4%）方面均显示出更佳的治疗反应，并且中位随访时间为35.2个月时，与克唑替尼相比，接受阿来替尼诱导治疗的患者的PFS明显更长（未达到 vs 17.9个月）。该研究表明在ALK融合阳性NSCLC患者新辅助治疗中，阿来替尼比克唑替尼表现出更优的疗效和长期益处。

上述研究表明，与新辅助化疗和免疫治疗相比，ALK-TKIs新辅助靶向治疗的MPR和pCR率有着显著的提升。尽管ALK融合阳性NSCLC的新辅助靶向治疗领域取得了一定的进展，但目前的研究数量相对较少，未涉及最新的第三代TKIs，且多为小规模、单中心的回顾性研究，缺乏多中心、前瞻性、随机对照的III期临床试验来提供更高级别的证据。期待后续临床研究数据的公布，或将成为可切除ALK融合阳性NSCLC患者新辅助靶向治疗的开端。

## 3 正在进行的ALK融合阳性新辅助靶向治疗研究

鉴于ALK-TKIs对ALK融合阳性NSCLC患者新辅助靶向治疗的积极疗效^[17,20]^及新一代ALK-TKIs在克服前代ALK-TKIs耐药性方面的显著效果^[21]^，目前关于ALK融合阳性NSCLC新辅助治疗的研究呈现出增长趋势。目前正在进行的ALK融合阳性NSCLC新辅助靶向治疗临床研究包括NAUTIKA1研究、ALNEO研究、NCT05361564、NCT05380024、NCT05765877、NCT06282536等。

NAUTIKA1研究^[22]^（NCT04302025）是一项正在进行的II期伞式研究，开始于2020年，预计2029年结束。该研究正在招募可切除IB-IIIA期或选择性IIIB期（仅T3N2）的ALK融合阳性NSCLC患者，该研究的主要终点为MPR。截至目前，共招募了12例符合条件患者，9例患者完成了阿来替尼新辅助治疗，有6例（66.7%）患者实现了MPR，其中3例（33.3%）实现了pCR，进行手术的8例患者均实现了R0切除。在NAUTIKA1的初步分析中，新辅助阿来替尼治疗表现出了良好的病理反应，在ALK融合阳性NSCLC患者中耐受良好，且没有患者因不良事件导致手术推迟。该研究入组仍在继续进行中。

另一项正在进行的研究为ALNEO研究^[23]^（NCT05015010），为一项II期、开放标签、单臂、多中心临床研究，旨在评估阿来替尼在III期未治疗的可能可切除（TxN2或T4N0-1）ALK融合阳性NSCLC患者中的应用。该研究的主要终点同样为MPR。在2024届世界肺癌大会（World Conference on Lung Cancer, WCLC）^[24]^上，研究者公布了相关数据。研究目前招募了25例患者，均完成了新辅助阿来替尼治疗，18例（86%）患者实行了R0切除，其中7例（39%）达到了MPR，3例（17%）达到了pCR。第一阶段中期分析的结果证明了新辅助阿来替尼对于可能可切除的局部晚期III期ALK融合阳性NSCLC患者的安全性与有效性，目前仍在招募患者入组，以达到总共33例计划患者。

其他尚未公布数据正在进行的临床研究包括NCT05361564、NCT05380024、NCT05765877、NCT06282536。NCT05361564为一项单臂、开放标签、II 期研究，该研究计划招募I-IIIA期患者。由于该研究目的为评估术前布加替尼的药物耐受持久性，所以pCR、MPR为其次要终点。该研究开始于2022年6月，预计2024年6月完成，但目前尚无数据公布。NEOEAST（NCT05380024）^[25]^为一项单臂、开放标签、前瞻性、II期研究，其目的是评估恩沙替尼作为可切除II-IIIB期ALK融合阳性NSCLC患者新辅助治疗的可行性。主要终点为MPR。该研究于2022年5月开始，目前正在进行中。NCT05765877、NCT06282536均为单臂探索性研究，且均使用伊鲁阿克进行干预。前者计划招募IB-IIIA期患者，主要终点为MPR，研究预计于2025年7月完成。后者则计划招募20例III-IVA期可能可切除的患者，其主要终点为客观缓解率（objective response rate, ORR），该研究预计于2029年12月完成。

由于ALK-TKIs的快速发展，目前进行的临床研究只使用第二代TKIs，未能运用新一代TKIs进行新辅助治疗的临床研究。这些研究中的临床实验设计相似，大多建议新辅助治疗时间为8周，术后辅助治疗时间为2年，主要终点为MPR。但不同研究中新辅助治疗与手术之间的间隔大不相同（3天到10周不等），这可能会影响患者新辅助治疗疗效与手术难度，仍需研究去进一步探索合适的手术时机。目前这些临床研究还在进行中，期待这些研究的最新数据能为之后ALK融合阳性NSCLC患者提供更好的治疗方案。

## 4 第一、二代ALK-TKIs新辅助治疗疗效比较

第一代ALK-TKIs克唑替尼的出现拉开了ALK阳性NSCLC靶向治疗的序幕。然而，由于其对血脑屏障的渗透率低，对中枢神经系统转移疗效较差^[26]^及获得性耐药，患者通常在用药1年内进展或死亡。相较之下，第二代ALK-TKIs具有更强的中枢系统疗效，并且展现出更优的治疗效果。因此对第一代与第二代ALK-TKIs新辅助治疗的疗效进行横向比较，有助于深入理解ALK-TKIs在新辅助靶向治疗中的进展。

在张超教授2019年的回顾性研究^[17]^与2023年AATS年会上的回顾性研究^[19]^中，新辅助克唑替尼治疗IIIA-IIIB期ALK融合阳性NSCLC的pCR率分别为18.2%和15.4%，淋巴结降期率分别为27.3%和45.5%。这些差异可能与样本量较小和新辅助靶向治疗的持续时间不同有关。

在第二代ALK-TKIs的临床研究中，SAKULA研究^[18]^采用塞瑞替尼新辅助治疗7例IIIA期ALK融合阳性NSCLC患者，实现了100%的ORR、57%的MPR以及29%的pCR。张超教授2023年AATS年会上的回顾性研究^[19]^中，20例IIIA-IIIB期ALK融合阳性NSCLC患者接受了新辅助阿来替尼治疗，MPR达到56.3%，pCR达到37.5%。NAUTIKA1研究^[22]^纳入了9例II-IIIB期ALK融合阳性NSCLC患者，采用新辅助阿来替尼治疗，MPR和pCR分别为66.7%和33.3%。ALNEO研究^[24]^中18例可手术切除的III期（TxN2, T4N0-1）ALK融合阳性NSCLC患者接受了新辅助阿来替尼治疗，MPR和pCR分别为38.9%和16.7%。

通过第一、二代ALK-TKIs新辅助治疗疗效对比，第二代ALK-TKIs在MPR和pCR方面均有显著提升，并且克服了第一代ALK-TKIs的耐药性问题。期待之后能够开展第三代ALK-TKIs新辅助治疗研究给患者带来更好的治疗方案。

## 5 讨论

ALK靶向治疗的出现彻底改变了ALK融合阳性NSCLC患者的治疗格局，但ALK新辅助治疗还存在诸多问题。鉴于ALK突变的罕见性和早期缺乏通用的基因检测，评估ALK-TKIs效果的大型随机研究的尝试受到了招募缓慢的挑战，既往数个相关临床研究都因招募失败而关闭，如SAKULA研究，该多中心研究自2015年3月至2018年3月共筛查了395例NSCLC患者，其中15例（4%）患者被确定为ALK融合阳性，最终仅招募了7例患者入组，这充分说明了ALK新辅助治疗临床研究的困难。此外，由于药物更新迭代迅速，许多正在进行的研究并没有使用最新一代的ALK-TKIs。

与此同时，在研究设计上还存在诸多问题需要解决。首先，关于ALK新辅助最佳治疗时间并未达成共识，治疗时间过短可能会无法达到R0切除，时间过长可能会导致肿瘤耐药性增加及手术难度增加。在先前的临床研究中ALK-TKIs作为新辅助治疗的使用尚未实现标准化的时间控制。例如，在两项回顾性研究^[17,19]^中，患者接受新辅助治疗的时间范围分别为28-120 d和80-145 d，这种显著的时间差异可能对治疗效果造成影响。目前大多数临床研究新辅助治疗时间为8周，既往研究^[17⇓-19]^已充分证明了ALK-TKIs新辅助治疗的安全性与有效性，增加新辅助治疗时间可能能够增加患者获益。SAKULA研究^[18]^纳入IIIA期ALK融合阳性NSCLC患者术前接受塞瑞替尼新辅助治疗12周，ORR达100%，因此本研究团队推荐ALK突变可切除的NSCLC新辅助治疗选用第三代ALK-TKIs，并将治疗时间延长至12周。此外，关于新辅助治疗后至手术的最佳时间间隔，现有文献中缺乏明确的指导。在张超教授2019年进行的回顾性研究^[17]^中手术间隔的中位时间为30 d，而在ALNEO研究^[23]^中其中位间隔时间为3周，NEOEAST研究^[25]^则报告了3-21 d的中位间隔时间。综合这些数据，目前对于新辅助靶向治疗与手术之间的最佳时间间隔尚无共识，不同的临床研究存在差异。这种时间间隔的变异性是否对患者的治疗效果有影响，仍需未来的研究来进一步验证。另外，术后辅助治疗的方案选择及其持续时间在学术界仍存在较大争议。NAUTIKA1和NEOEAST研究^[22,25]^采用辅助化疗4个周期后继以2年的辅助靶向治疗的策略。相比之下，ALNEO^[23]^、NCT05765877和NCT06282536研究则采用了单纯的2年辅助靶向治疗。目前的临床研究普遍倾向于采用辅助靶向治疗。尽管化疗具有一定的疗效，但其伴随的不良反应较为严重。综上，本研究团队推荐术后靶向治疗时间为2年及以上，但是否需要将化疗纳入辅助治疗以增强疗效的问题仍需进一步的研究探讨和证据支持。最后，对于肿瘤临床研究来说OS是其主要终点，但从入组到新辅助研究OS数据发表通常需要10多年的时间且需要巨大的人力物力资源，这需要强有力的替代指标来加速新辅助治疗的研究。目前国际推荐采用MPR（即切除的肺和淋巴结组织中≤10%的残留肿瘤组织）作为OS的替代指标^[27]^，然而在接受新辅助靶向治疗的可切除NSCLC患者中OS与MPR之间的关系尚未完全阐明。一项新辅助吉非替尼II期研究^[28]^表明，MPR与DFS相关，但与OS无关。同样在一项荟萃分析^[29]^中研究者评估了MPR能否作为早期可切除NSCLC无事件生存期（event-free survival, EFS）和OS的替代终点，得出MPR与2年EFS之间存在稳健的相关性但与OS无关的结论。目前，本研究团队仍然推荐将MPR作为评估ALK融合阳性NSCLC新辅助靶向治疗的主要终点指标。采用MPR作为主要终点能够减少临床研究所需的时间和资源消耗。然而，为了确立MPR与OS之间的相关性，还需要更多的数据来明确MPR与OS之间的关联。

## 6 总结

本文综述了目前ALK融合阳性NSCLC新辅助靶向治疗的进展，对于可切除ALK融合阳性NSCLC患者，新辅助靶向治疗可显著缩小肿瘤体积，提高根治性手术切除率及增加病理缓解率，但仍存在以下诸多问题需要解决：（1）如何提高ALK融合阳性NSCLC患者检出率；（2）新辅助靶向治疗最佳治疗时间应为多久；（3）术后辅助靶向治疗联合化疗是否优于辅助靶向治疗；（4）MPR作为新辅助靶向治疗的主要终点能否准确反映患者OS。未来，随着NAUTIKA1、ALNEO、NEOEAST等研究结果的公布，我们期待能够解答以上问题，为ALK融合阳性可切除NSCLC患者提供更好的新辅助靶向治疗策略。

## References

[1] Siegel RL, Giaquinto AN, Jemal A. Cancer statistics, 2024. CA Cancer J Clin, 2024, 74(1): 12-49. doi: 10.3322/caac.21820 38230766

[2] Goldstraw P, Chansky K, Crowley J, et al. The IASLC lung cancer staging project: proposals for revision of the TNM stage groupings in the forthcoming (eighth) edition of the TNM classification for lung cancer. J Thorac Oncol, 2016, 11(1): 39-51. doi: 10.1016/j.jtho.2015.09.009 26762738

[3] Wakelee H, Liberman M, Kato T, et al. Perioperative pembrolizumab for early-stage non-small cell lung cancer. N Engl J Med, 2023, 389(6): 491-503. doi: 10.1056/NEJMoa2302983 37272513 PMC11074923

[4] He J, Gao S, Reck M, et al. OA12.06 Neoadjuvant durvalumab+ chemotherapy followed by adjuvant durvalumab in resectable EGFR-mutated NSCLC (AEGEAN). J Thorac Oncol, 2023, 18(11 supplement): S72-S73. doi: 10.1016/j.jtho.2023.09.071 40545237

[5] Forde PM, Spicer J, Lu S, et al. Neoadjuvant nivolumab plus chemotherapy in resectable lung cancer. N Engl J Med, 2022, 386(21): 1973-1985. doi: 10.1056/NEJMoa2202170 35403841 PMC9844511

[6] Shackelford RE, Vora M, Mayhall K, et al. ALK-rearrangements and testing methods in non-small cell lung cancer: a review. Genes Cancer, 2014, 5(1-2): 1-14. doi: 10.18632/genesandcancer.3 24955213 PMC4063252

[7] Solomon BJ, Liu G, Felip E, et al. Lorlatinib versus crizotinib in patients with advanced ALK-positive non-small cell lung cancer: 5-year outcomes from the phase III CROWN study. J Thorac Oncol, 2024, 42(29): 3400-3409. doi: 10.1200/JCO.24.00581 PMC11458101 38819031

[8] Solomon BJ, Mok T, Kim DW, et al. First-line crizotinib versus chemotherapy in ALK-positive lung cancer. N Engl J Med, 2014, 371(23): 2167-2177. doi: 10.1056/NEJMoa1408440 25470694

[9] Soria JC, Tan DSW, Chiari R, et al. First-line ceritinib versus platinum-based chemotherapy in advanced ALK-rearranged non-small-cell lung cancer (ASCEND-4): a randomised, open-label, phase 3 study. Lancet, 2017, 389(10072): 917-929. doi: 10.1016/S0140-6736(17)30123-X 28126333

[10] Wu YL, Dziadziuszko R, Ahn JS, et al. Alectinib in resected ALK-positive non-small cell lung ccancer. N Engl J Med, 2024, 390(14): 1265-1276. doi: 10.1056/NEJMoa2310532 38598794

[11] Gainor JF, Shaw AT, Sequist LV, et al. EGFR mutations and ALK rearrangements are associated with low response rates to PD-1 pathway blockade in non-small cell lung cancer: a retrospective analysis. Clin Cancer Res, 2016, 22(18): 4585-4593. doi: 10.1158/1078-0432.CCR-15-3101 27225694 PMC5026567

[12] Soda M, Choi YL, Enomoto M, et al. Identification of the transforming EML4-ALK fusion gene in non-small cell lung cancer. Nature, 2007, 448(7153): 561-566. doi: 10.1038/nature05945 17625570

[13] Ou SI, Zhu VW, Nagasaka M. Catalog of 5’ fusion partners in ALK-positive NSCLC circa 2020. JTO Clin Res Rep, 2020, 1(1): 100015. doi: 10.1016/j.jtocrr.2020.100015 PMC8474466 34589917

[14] Ando K, Manabe R, Kishino Y, et al. Comparative efficacy and safety of lorlatinib and alectinib for ALK-rearrangement positive advanced non-small cell lung cancer in Asian and non-Asian patients: A systematic review and network meta-analysis. Cancers (Basel), 2021, 13(15): 3704. doi: 10.3390/cancers13153704 PMC8345181 34359604

[15] Hallberg B, Palmer RH. The role of the ALK receptor in cancer biology. Ann Oncol, 2016, 27 Suppl 3: iii4-iii15. doi: 10.1093/annonc/mdw301 27573755

[16] Johnson TW, Richardson PF, Bailey S, et al. Discovery of (10R)-7-amino-12-fluoro-2,10,16-trimethyl-15-oxo-10,15,16,17-tetrahydro-2H-8,4-(metheno)pyrazolo[ 4,3-h][2,5,11]-benzoxadiazacyclotetradecine-3-carbonitrile (PF-06463922), a macrocyclic inhibitor of anaplastic lymphoma kinase (ALK) and c-ros oncogene 1 (ROS1) with preclinical brain exposure and broad-spectrum potency against ALK-resistant mutations. J Med Chem, 2014, 57(11): 4720-4744. doi: 10.1021/jm500261q 24819116

[17] Zhang C, Li SL, Nie Q, et al. Neoadjuvant crizotinib in resectable locally advanced non-small cell lung cancer with ALK rearrangement. J Thorac Oncol, 2019, 14(4): 726-731. doi: 10.1016/j.jtho.2018.10.161 30408570

[18] Zenke Y, Yoh K, Sakakibara-Konishi J, et al. Neoadjuvant ceritinib for locally advanced non-small cell lung cancer with ALK rearrangement:SAKULA trial. J Thorac Oncol, 2019, 14(10 supplement): S626-S627. doi: 10.1016/j.jtho.2019.08.1320

[19] Zhang C, Liu SY, Jiang BY, et al. Induction ALK-TKIs for stage III non-small cell lung cancer harboring ALK fusion:a single-center experience with 3-year follow-up. Presented at the 103^rd^ Annual Meeting of the American Association for Thoracic Surgery, Los Angeles, CA. https://www.aats.org/resources/induction-alk-tk-is-for-stage-iii-non-small-cell-lung-cancer-harboring-alk-fusion-a-single-center-experience-with-3-year-follow-up.https://www.aats.org/resources/induction-alk-tk-is-for-stage-iii-non-small-cell-lung-cancer-harboring-alk-fusion-a-single-center-experience-with-3-year-follow-up Accessed Oct 15, 2024

[20] Zhang C, Yan LX, Jiang BY, et al. Feasibility and safety of neoadjuvant alectinib in a patient with ALK-positive locally advanced NSCLC. J Thorac Oncol, 2020, 15(6): e95-e99. doi: 10.1016/j.jtho.2019.12.133 32471573

[21] Horn L, Infante JR, Reckamp KL, et al. Ensartinib (X-396) in ALK-positive non-small cell lung cancer: results from a first-in-human phase I/II, multicenter study. Clin Cancer Res, 2018, 24(12): 2771-2779. doi: 10.1158/1078-0432.CCR-17-2398 29563138 PMC6004248

[22] Lee JM, Toloza EM, Pass HI, et al. NAUTIKA1 study: Preliminary efficacy and safety data with neoadjuvant alectinib in patients with stage IB-III ALK+ NSCLC. J Thorac Oncol, 2023, 18(11 supplement): S297-S298. doi: 10.1016/j.jtho.2023.09.511

[23] Leonetti A, Minari R, Boni L, et al. Phase II, open-label, single-arm, multicenter study to assess the activity and safety of alectinib as neoadjuvant treatment in surgically resectable stage III ALK-positive NSCLC: ALNEO trial. Clin Lung Cancer, 2021, 22(5): 473-477. doi: 10.1016/j.cllc.2021.02.014 33762169

[24] Leonetti A, Boni L, Gnetti L, et al. MA01.03 Neoadjuvant alectinib in potentially resectable stage III ALK-positive NSCLC: Interim analysis of ALNEO-GOIRC-01-2020 phase II trial. J Thorac Oncol, 2024, 19(10): S52. doi: 10.1016/j.jtho.2024.09.091

[25] Lu FL, Lv C, Zhuo ML, et al. EP07.05-02 NEOEAST: a phase II study of ensartinib as neoadjuvant therapy for stage II-IIIB ALK-rearranged non-small cell lung cancer. J Thorac Oncol, 2023, 18(11 supplement): S558. doi: 10.1016/j.jtho.2023.09.1045

[26] Costa DB, Kobayashi S, Pandya SS, et al. CSF concentration of the anaplastic lymphoma kinase inhibitor crizotinib. J Clin Oncol, 2011, 29(15): e443-e445. doi: 10.1200/JCO.2010.34.1313 21422405

[27] Hellmann MD, Chaft JE, William WN Jr, et al. Pathological response after neoadjuvant chemotherapy in resectable non-small-cell lung cancers: proposal for the use of major pathological response as a surrogate endpoint. Lancet Oncol, 2014, 15(1): e42-e50. doi: 10.1016/S1470-2045(13)70334-6 PMC4734624 24384493

[28] Zhang Y, Fu F, Hu H, et al. Gefitinib as neoadjuvant therapy for resectable stage II-IIIA non-small cell lung cancer: A phase II study. J Thorac Cardiovasc Surg, 2021, 161(2): 434-442.e2. doi: 10.1016/j.jtcvs.2020.02.131 32340810

[29] Hines JB, Cameron RB, Esposito A, et al. Evaluation of major pathologic response and pathologic complete response as surrogate end points for survival in randomized controlled trials of neoadjuvant immune checkpoint blockade in resectable in NSCLC. J Thorac Oncol, 2024, 19(7): 1108-1116. doi: 10.1016/j.jtho.2024.03.010 38461929 PMC12697759

